# Fitting in an Unfit Society With Autism Spectrum Disorder: Case Report

**DOI:** 10.7759/cureus.36966

**Published:** 2023-03-31

**Authors:** Kathleen A Morrisroe, Katherine Longo, Patricia Pebley, Lakshit Jain

**Affiliations:** 1 Department of Psychiatry, University of Connecticut School of Medicine, Farmington, USA; 2 Department of Psychology, Graduate Institute of Professional Psychology, University of Hartford, Hartford, USA; 3 Department of Psychology, Connecticut Valley Hospital, Middletown, USA; 4 Department of Psychiatry, University of Connecticut, Hartford, USA

**Keywords:** psycho-social intelligence, social psychiatry, social networks, inpatient psychiatry services, social constructivist learning environment, case report, self harm, camouflaging, behavioral mimicking, autism spectrum disorder

## Abstract

Evaluating behavioral mimicking is important in healthcare providers' everyday functioning with an increased presentation of Tourette syndrome-like cases during the COVID-19 pandemic, seen due to the popular video creators on social media (e.g., TikTok) exhibiting these behaviors. Individuals with autism spectrum disorder (ASD) face difficulties with connection and assimilation, and they adapt by camouflaging their behaviors to fit with those of the neurotypical majority. Our team evaluated the behaviors of one individual with ASD to establish whether camouflaging was playing a role in her psychiatric stabilization in our inpatient psychiatric unit. We present a case of a 30-year-old female with ASD, admitted to our long-term inpatient psychiatric facility for significant mood dysregulation that persisted despite numerous treatment approaches (mediations, groups, etc.). While her initial behaviors included head banging and self-induced falls, her behaviors seemed to change based on those of her peers, in an apparent attempt to camouflage into the social environment within the unit. She also appeared to learn new self-harm behaviors, such as skin picking, from peers around her. The team was able to establish a temporal link between some instances of peers exhibiting specific behaviors and our patient engaging in similar behavior. Although inpatient units effectively manage long-term stabilization in other psychiatric disorders, these environments are not designed for individuals with ASD. Treatment teams should recognize the malleability of behaviors in patients with ASD and must identify and manage behavioral mimicking early during inpatient psychiatric treatment; otherwise, it may lead to significant harm.

## Introduction

Human beings are social animals. We desire to be part of a group of people who care for us [1]. The COVID-19 pandemic brought with it social isolation that greatly impacted societies across the globe [2]. Amid this isolation, there was an explosion in social media use by children and adolescents [3]. This included apps such as TikTok, where they watched several popular creators, with some creators exhibiting symptoms consistent with Tourette’s syndrome (TS). After these videos “went viral,” there was a marked increase in tic-like mannerisms in viewers leading to increased visits to physician offices [4]. However, many tics exhibited by these viewers were not of the type that is normally seen in TS [4]. For example, there was no fluctuation in the tics, they were often goal-directed and context-dependent, and there were far more incidences of coprolalia, and self-injurious behavior [3,4]. This display of behavioral mimicking by those viewing social media seems primarily driven by a desire for connection and assimilation [4]. Olvera et al. describe this as an “example of mass sociogenic illness,” largely stemming from behavioral mimicking due to the environment of isolation [3]. Other researchers term this a “somatic stress response”. There are examples of this throughout history including epidemics of paralysis, stuporous states, and tremors in periods following the French Revolution, Napoleonic Wars, and World War I [5].

Similarly, those with autism spectrum disorder (ASD) utilize mimicking in order to fit into the social environment around them [4]. The Diagnostic and Statistical Manual (DSM) 5 criteria for ASD include social communication and interaction deficits across multiple domains, such as conversation, nonverbal communication, and difficulty with relationships, that are present in early development and cause significant impairment in functioning [6]. Also seen are repetitive patterns of behavior and interests, such as rigid routines, hyper- or hypo-reactivity, and stereotyped movements [6]. People with ASD have a reduced ability to manage a social situation, to understand how they are perceived among peers and hence are more likely to adopt behaviors from their surroundings [7,8]. As a strategy to cope with situations in which they feel different from the neurotypical majority, people with ASD sometimes hide behaviors associated with their diagnosis using techniques termed “camouflaging” [7,9]. One person described camouflaging as an attempt to “people suit… as if playing a game,” that is done “on autopilot” because it becomes ingrained in their identity [9]. Questionnaires developed for evaluating camouflaging rates and behaviors (like Camouflaging Autistic Traits Questionnaire (CAT-Q)) estimate that around 90% of people with ASD report camouflaging their ASD-related behaviors to fit in with society. Noticeably, females scored higher on the questionnaire overall [10-12]. Motivations behind camouflaging include strengthening relationships and compensating for cognitive difficulties encountered in daily life [7,11]. While camouflaging does allow people to fit into social environments more effectively, it does not come without risk. One study found that the exhaustion from trying to adopt behaviors that one does not understand and are not their own as well as the anxiety regarding what one should be doing in different instances is an independent risk factor for suicidality in this population [7,10].

A behavior that many try to camouflage is self-stimulating behavior, or “stimming.” Up to 72% of patients describe using these behaviors, such as hand-flapping, body-rocking, or pacing, to reduce anxiety and cope with overstimulation [13,14]. Many attribute stimming to an inability to process feelings of overwhelming emotion, which leads to a need to expel the sensation through an action [13,14]. Most are not self-injurious, although some can cause harm unintentionally (head banging, self-biting, skin-scratching, etc.) [8,15]. Although 42% of patients engage in intentional self-harm, there has been no association found between the degree of intellectual disability and self-injury. This indicates that many of these behaviors may be learned [8,15]. In this paper, we present the case of S, who is a patient with ASD and has a history of self-harm and stimming behaviors. These behaviors continued despite her stay at the inpatient psychiatric unit and at times morphed into self-harm behaviors that were similar to other patients on the unit. This was likely due to her camouflaging and trying to fit into the social environment of an inpatient psychiatric unit.

## Case presentation

Our patient (S) is a 30-year-old woman with ASD and borderline/mild intellectual disability, diagnosed at age nine. S was born full-term at average birth weight after an uncomplicated pregnancy. She was hypotonic at birth and experienced early developmental delays, leading to diagnoses of myopia, expressive language delays, and fine motor and oral motor delays as a toddler. She received Birth-to-Three services and attended a special education preschool program. Records indicate that she had visual-motor and coordination difficulties, oppositional behavior, and difficulty getting along with peers. A correlation between poor communication and aggression was established early, with social issues that started in preschool and persisted throughout her schooling. These included "tantrums" that lasted up to two hours and escalation to physical aggression. This led to multiple expulsions, and she was transferred from public schools to therapeutic day schools, residential placements, and then back to public high school. From age 10 to 19, S required at least eight psychiatric hospitalizations for physical aggression toward others, homicidal and suicidal ideation, and mood dysregulation across multiple settings. This was all despite numerous treatment approaches. She had a history of foreign body ingestion (coins), but she was observed not to engage in it unless others exhibited it around her. She had at least two suicide attempts, one of which was a pill ingestion that resulted in a month-long medical hospitalization. After her discharge, she was transferred to a psychiatric hospital for stabilization and then was transitioned to an intensive residential program. While at the intensive residential program, S demonstrated a period of relative stability for about seven years. During this time, she was able to transition into a therapeutic foster home and attend day treatment, eventually leading to a vocational program. Unfortunately, when S was transitioned from the therapeutic foster home to another residential program (that was closer to her mom’s home), S exhibited episodes of property destruction and self-harm that required physical restraints and psychiatric hospitalizations. She was admitted to our inpatient unit after several attempts to stabilize her at short-term hospitals were unsuccessful.

During her stays at various institutions across 20 years prior to coming to our unit, the patient had numerous assessments that demonstrated significant variability in regard to her psychiatric diagnosis and cognitive functioning. This likely reflects an inconsistent ability to maintain effort and attention. Her varied cognitive ability and inconsistent effort, coupled with her difficulty in coping with emotional demands, negatively impact her ability to demonstrate functioning at an average cognitive level. In these assessments, S consistently demonstrated difficulty with impulsivity, sustained attention, restlessness, and poor frustration tolerance. In addition, the rate at which she processes, encodes, integrates, and learns both novel and routine information is delayed. Emotional functioning assessments found that S experiences rapid shifts in self-definition and behavioral volatility when faced with frustration or interpersonal disagreements. S is impaired in her ability to form accurate impressions of the intentions of others, particularly when the situation becomes unfamiliar, less structured, or more emotionally charged. Profound confusion and disorganized thinking prevent S from forming effective interpersonal connections. S is reactive to small stimuli and likely has a tendency to focus on her needs at the expense of others. All of these factors lead to impulsive behaviors that put her at high risk for self-harm and suicidality.

Hospital stay

Our facility is a long-term inpatient state psychiatric hospital with patients being admitted for several months to years. Therefore, many develop friendships and cliques over time. We have a “level” system, which determines the level of independence and privileges that a patient receives based on their clinical stability. For example, patients placed on close observation (CO) status for either psychiatric or medical concerns require consistent direct supervision by staff to ensure safety. Patients with higher levels have privileges like the ability to go outside the unit for walks for up to one hour.

The first month of S’s admission was spent on a unit different from that of the authors. During this month, peers on the unit targeted her and S engaged in frequent concerning behaviors including an episode of head banging. S then experienced a cluster of falls that appeared self-induced, followed by an assault, and several episodes of head banging, with the average time between episodes being around two days.

After her transfer to our unit, S had no episodes for the first three weeks. S was not necessarily welcomed into specific groups on her arrival to the unit. It became clear that she wanted to connect with two of her female peers (M and C) who were on CO. In addition, a third female peer (P) was bullying S. In the majority of cases, patients tend to want more independence and request higher levels; however, S repeatedly requested CO rather than independence, perhaps as a way to appear similar to peers who were on CO or to seek protection from bullying. Our team noted that S engaged in head banging or banging her feet and elbows as stimming but did not injure herself by engaging in these behaviors. In most cases, S was successful in regulating her emotions through these techniques, although when these behaviors were interrupted or not successful, she was at an increased risk of assaultive or self-injurious behavior.

As the dates indicate, S's hospital stay was complicated by the COVID-19 pandemic. The inpatient unit was placed on quarantine several times, and almost all staff and patients contracted COVID-19 at least two times (including S). S was effectively cut off from her "society" as she was unable to meet her mother in person. As her mother did not use an iPhone, she was unable to engage in FaceTime with S. S was also terrified of her mother contracting COVID-19 as she had several medical comorbidities (hypertension, diabetes, and cancer) that would have severely impacted her survival. S was also able to watch TV news and would keep tabs on the morbidity and mortality caused by COVID-19. 

Over the next few days, a pattern started to develop, which we highlight in Table 1. Actions of M, C, and P led to a similar behavior or a reaction from S (at times immediately and at times several days after). M and C engaged in self-harm behaviors that led them to be placed on CO, and this appeared to stimulate S’s desire to be on CO as well. P was directly aggressive toward S, and that led to a desire for S to be placed on CO for safety and/or a visit to ER to get off the unit. We can also see an example of S using head banging as stimming to mask her frustration with peers on 2/4, and its failure leading to aggression on 2/5.

**Table 1:** Behavioral patterns of S and their temporal correlation with behaviors of other peers S: our patient; M and C: female peers who engage in self-harm behaviors and S wants to be friends with; P: female peer who bullied S; CO: close observation; RN: registered nurse; SI: suicidal ideation; FB, foreign body.

Date	Behavior	Surrounding behaviors
1/13/21	Head banging	1/12: P hit a male peer as staff were attempting to de-escalate a verbal argument between them. 1/13: C attacked a male peer who was antagonizing other female patients
1/21/21	Fall	1/15: M reported she had been trying to pull screws off the wall in the day-hall and planned to cut herself
2/4/21	Head banging	2/5: P lunged at S and attempted to assault her because S called P fat
2/25/21	Head banging	2/11: M swallowed a pen and was sent to the ER. 2/16: M returned from the hospital and was agitated, threatening to harm staff and self. 2/17: M attempted to steal a flashlight from CO staff
3/19/21	Assault	3/19: P reported to the RN and several peers “I want to die. I want to kill myself. I am not safe.”
4/16/21	Assault	4/9: C attempted to self-harm for several days. She was placed in restraints.
4/20/21	Head banging	4/17: P made verbal threats to S, “I don’t care. I will kick her ass; I’m going to fuck her up.”
5/5/21	SI	4/20: M swallowed metal buttons and pieces of a mechanical pencil in an attempt to self-harm
5/10/21	Assault	5/2: P assaulted S
5/24/21	Head banging	5/6: C scratched her arm, verbalized SI and that she misses her deceased mother
7/6/21	Banging elbows and fists	6/23: M reported she saw a male peer hoarding cigarettes, then had a verbal argument with him
8/2/21	SI	7/2: P spit at S, verbalizing “I’m not gonna calm down” and “I want to hit someone”. 7/3: P was observed lying on the floor. She had no injuries and refused to get up.
9/10/21	Head banging	7/27: M ingested multiple objects after complaining about a female peer
9/22/21	Fall	9/4: M swallowed her eyeglass frames. 9/8: M accused the social worker of ignoring her and reported feeling “like a failure” after the ingestion
9/27/21	Banging elbows and fists	9/21: P fell while getting out of the shower. Witnessed fall, no injury.
10/4/21	Assault	9/25: RN confiscated several objects that M was in possession of that she had recently swallowed
11/29/21	Head banging	9/29: M returned from ER after ingestion of multiple foreign objects and was placed on CO. Over the next two months, she engaged in several FB ingestion behaviors

We were not able to establish causation, as there were behaviors S engaged in that had no co-occurring peer behaviors. These include four episodes of fall on 1/23, 1/24, 1/28, and 6/24; an assault on 2/1; head banging on 2/3 and 11/29; and banging elbows and fists on 3/1. In addition, there was an episode involving a peer in a different unit. In this episode, S reported an abrasion to her left elbow at the end of January, which due to her repeated scratching turned to cellulitis, and subsequently sepsis, leading to emergency surgery and a brief medical stay. After returning to the unit, she refused to walk and would only use the wheelchair in an apparent attempt to be placed on CO for fall risk. She also began unwrapping the wound dressing and inserting her finger into the wound. For the following two weeks, she continued to pick at the wound despite several discussions with different team members and family. There had been no prior reports of S exhibiting picking behaviors in the past. Notably, the same behavior has been the self-harm method of choice of a female peer (D) on a different unit (picking at and inserting objects in wounds). S reported to the team that she had frequent telephone conversations with D (and may have been taught to do this behavior by D) and declared her a friend.

These behaviors appear to represent mimicking behavior, as the patient attempts to acclimate to her social environment. The differential diagnosis for these behaviors includes Cluster B personality disorder. S exhibits affectivity and difficulty with interpersonal functioning, although it seems as though this pattern was first noted within the inpatient psychiatric facility, and it would be unlikely for a personality disorder to develop this late in life. Another differential diagnosis could be malingering, as the patient may have been seeking secondary gain from staff or other patients. Conversion disorder is also a possible differential, although we might expect more discrete neurologic symptoms if this were the case. Finally, the behaviors may be products of normal feelings of jealousy and anger. However, based on the proximity to the other behaviors that she had witnessed, this seems less likely.

Throughout her stay, she was trialed on many psychiatric medications. These include trials of topiramate, olanzapine, diazepam, and ativan, which were not effective in regulating her behaviors. She is currently psychiatrically stable on clozapine (50 mg in the morning and 350 mg at night by mouth), valproic acid delayed-release (DR) (750 mg in the morning and every (Q) 8 PM by mouth), glycopyrrolate (2 mg in the morning, at 11 AM, and at 2 PM by mouth for clozapine-induced drooling), escitalopram (20 mg in the morning by mouth), propranolol long-acting (LA) (120 mg in the morning by mouth), and bupropion extended-release (XL) (450 mg in the morning by mouth). There were multiple attempts at non-pharmacological therapeutic interventions, such as verbal redirection, as well as education of staff working with S to prevent caregiver fatigue. Additionally, staff work with S to develop relationships, so she has healthier alternative behaviors to mimic with group activities including playing music or doing puzzles. With our efforts, the team was able to increase the average time between episodes from 2 to 22 days with two occasions of 56 days long behavior-free periods. S continues to do well at the submission date of this case report and is currently engaging in discharge planning.

Unfortunately, S is likely to remain hospitalized due to her continued aggression toward herself, others, and property when she becomes emotionally dysregulated. Her prognosis is moderate to poor, and she is considered gravely disabled as she is unlikely to be able to fend for herself without adequate support and supervision as evidenced by her behaviors. She was able to reside in a group home for four years before her condition deteriorated, and hence once stabilized appropriately, she will likely benefit from discharge to a group home near her mother’s place of residence.

## Discussion

While it is difficult to establish causation between S’s behaviors and the behaviors of peers on the unit, there does seem to be an association with the behaviors of female peers in the milieu. Additionally, her response to picking at her cellulitis sparked interest in this topic because there has been no record of her exhibiting skin-picking behaviors in the past. It is feasible to hypothesize that many of S’s actions are her “camouflaging” to better fit in with the social environment of which she is currently a part of (the inpatient psychiatric unit) [7].

Despite difficulty with social interaction, people with ASD desire connection, and S’s requests for CO status and requests to go to the ER may represent attempts at making social connections [7]. While there may be some component of a fixed obsession with somatic symptoms (possibly related to family illnesses and deaths in her formative years), this appears to be a learned behavior as it often supplied her with an increased level of care and more attention from those around her. The “compensation hypothesis” states that those with cognitive difficulties will use alternative neural routes and psychological strategies in order to compensate for their disability [11]. She may also be using other somatic concerns and behaviors in order to have more people around her regularly [11]. Our team was able to tackle these requests through additional one-on-one sessions with her in order to allay this anxiety. The writer also engaged with the on-call providers to make sure that these strategies were replicated by them as well.

General psychiatric hospital environments have not adapted to the unique needs of ASD pediatric patients, and the same is likely true of adults [16]. The constant change in staff, procedures, and the environment (e.g., when S was moved to a different inpatient psychiatric unit within one month of her admission to our facility) leads to anxiety, which can cause disruptive behaviors [16]. There are only nine specialty ASD inpatient programs in the United States in 2012 despite reports that when staff are specifically trained in working with ASD patients, there is a decreased use of restraints on the unit, as well as a decrease in the PRN (as needed) medication administration [16]. One diagnosis that we considered S met the criteria for was Social Breakdown Syndrome. This is seen in chronic mental health patients as the lack of positive reinforcement while hospitalized leads to a decrease in social and occupational skills. This often leads to drastic changes in behaviors [17]. With a lessened ability to respond positively to reinforcement on inpatient psychiatric units, a patient like S is more likely to lose some of their appropriate community behaviors. By employing camouflaging, patients are able to adapt their behavior accordingly [7,16]. Due to the nature of the environment, the majority of reinforcement from staff tends to come from a withdrawal of privileges for explosive behaviors (e.g., a drop in level or being placed on CO), with very little reward for everyday activities such as grooming [17]. As seen in this case study, it appears that this patient’s primary interest was in obtaining the attention of those around her, whether positive or negative, and therefore, she was able to adjust her behavior accordingly. By identifying this trend in our patient’s behaviors, there may be an opportunity to use behavioral mimicking as a means of teaching new behaviors deemed appropriate and beneficial by the treatment team. For example, whether the patient has ASD or is neurotypical, modeling “normal” behavior, would give patients an opportunity to mimic this behavior in a positive way that helps their treatment outcomes.

There are significant limitations to this case report, as we observed the behavior of one patient in one unit. Additionally, as described throughout our differential diagnoses, the patient has several comorbidities that may be influencing her behaviors. We selected this patient based on the observations of her behavior as it related to others’ behaviors on the unit; therefore, there is a component of selection bias. In addition, there is a substantial body of research in the field of social and behavioral health as well as sociological literature indicating how the social environment of the mental hospital can produce mental disorders, which the authors were not initially familiar with [18-20].

## Conclusions

Although we were unable to establish causation, it appears as though S is modifying her behavior in concordance with the patients around her in order to assimilate in this new social environment caused by frequent COVID-19 pandemic-induced quarantines. Integration of camouflaging into a behavioral model in the inpatient setting should be an important consideration in building treatment plans. Since patients with ASD are prone to this influence, it is important to consider the effectiveness of general psychiatric hospitalization in this population. For those with ASD, significant efforts should be made to limit the length of hospitalization, as it is possible for patients to learn concerning behaviors like self-harm. There has been benefit noted in specialty ASD inpatient programs, and the need of the hour is to replicate these learning in inpatient psychiatric treatment centers across the country. The incorporation of positive reinforcement as a tool to motivate inpatient psychiatric patients to choose healthy behaviors is needed. Human beings desire to be part of a community, and this desire continues for those who suffer from disorders of social communication such as ASD. Especially in the presence of mass social stressors that interrupt communications (pandemics like COVID-19 and wars), people are able to shift their behavior and adapt to new challenges. For S, admission to the long-term psychiatric facility and integration into her newfound community was likely a large stressor and the COVID-19 pandemic-induced quarantines further complicated this. Therefore, she seemed to adapt to the behaviors of those around her to become a part of this "society". These behavioral shifts are based on communication and setting. Clinicians should be cognizant of how they affect our patients and must adjust accordingly to the ever-changing nature of both of those variables.
